# Analysis of risk factors and construction of nomogram model for enteral nutrition-related diarrhea in ICU patients

**DOI:** 10.12669/pjms.42.1.12838

**Published:** 2026-01

**Authors:** Demei Zhao, Tingting Chen, Yanhua Hu, Qiongqiong He, Siyu Ji

**Affiliations:** 1 Demei Zhao Department of Intensive Care Medicine, Shanghai Blue Cross Brain Hospital, Shanghai 201101, P.R. China; 2 Tingting Chen Neurological Intensive Care Unit (NICU), Shanghai Blue Cross Brain Hospital, Shanghai 201101, P.R. China; 3 Yanhua Hu Department of Neurology, Shanghai Blue Cross Brain Hospital, Shanghai 201101, P.R. China; 4 Qiongqiong He Neurological Intensive Care Unit (NICU), Shanghai Blue Cross Brain Hospital, Shanghai 201101, P.R. China; 5 Siyu Ji Department of Intensive Care Medicine, Shanghai Blue Cross Brain Hospital, Shanghai 201101, P.R. China

**Keywords:** Enteral nutrition, Intensive care unit, Nomogram model, Nursing, Risk factors

## Abstract

**Objective:**

To analyze risk factors of enteral nutrition (EN) diarrhea in intensive care unit (ICU) patients and to construct nomogram model.

**Methodology:**

A retrospective analysis was conducted on 402 patients who received EN treatment in the ICU of Shanghai Blue Cross Brain Hospital from January 2022 to January 2025. They were divided into diarrhea group and non-diarrhea group based on the occurrence of EN diarrhea. We used univariate and multivariate logistic regression analysis to identify risk factors for EN diarrhea. Construct a nomogram model for the occurrence of EN diarrhea in ICU patients based on independent risk factors and conduct goodness of fit tests.

**Results:**

The incidence of EN diarrhea in ICU patients was 29.85% (120/402). Hypoproteinemia, abdominal surgery in the last five days, fasting time > 5 days, antibiotic use > 2 weeks, no gradual increase in EN preparations, oral potassium preparations, EN liquid infusion rate ≥ 100ml/h, albumin level ≥ 35g/L were identified as risk factors for EN diarrhea (p<0.05). Based on the above influencing factors, a nomogram model was constructed and internally validated using the Bootstrap method. The nomogram model basically fitted with the ideal model. ROC area under curve (AUC) was 0.860 (95% CI: 0.820-0.901), indicating a certain predictive value for EN diarrhea.

**Conclusions:**

The nomogram model has high predictive power in predicting the risk of EN diarrhea in ICU patients. It is beneficial for screening high-risk patients and further adjusting nutritional therapy.

## INTRODUCTION

Intensive care unit (ICU) patients generally present with severe conditions and are often unable to receive oral nutrition,^1^ have high prevalence of energy consumption and protein breakdown, and are in a state of high decomposition and metabolism.^2^ Therefore, these patients require nutritional support therapy, such as EN, to maintain their nutritional needs. While providing nutritional support, such therapy can promote proper immune function, tissue recovery and healing.^3^

However, since ICU patients are critically ill, EN treatment is often associated with a risk of multiple complications. EN diarrhea is a common adverse effect of nutritional therapy, with an incidence of 20% to 40%.^4^ This complication may lead to the increased loss of water and electrolytes, and malnutrition, and is associated with higher mortality.^4^,^5^ Therefore, identifying risk factors of EN diarrhea in ICU patients for timely prevention of this adverse effect is crucial.^3^-^5^

Recent studies have found that the risk factors for EN diarrhea include incorrect EN dosage and infusion rate, use of antibiotics, and longer fasting periods.^6^ However, there is a substantial variability in the risk factors among different studies.^6^,^7^ Current study aimed to analyze clinical data of 402 patients who received EN treatment to identify risk factors of EN diarrhea, and to create a nomogram model that may be used in clinical practice.

## METHODOLOGY

Clinical data of 402 ICU patients who received EN in Shanghai Blue Cross Brain Hospital from January 2022 to January 2025 were retrospectively selected. Patients were divided into the diarrhea group (n=120) and a non-diarrhea group (n=382) based on whether they experienced diarrhea during EN treatment in the ICU.

### Ethical approval:

The ethics committee of Shanghai Blue Cross Brain Hospital approved the study with the number BCEC-2025-06-0707; dated July 7, 2025.

### Inclusion criteria:

- Admission to the ICU and implementation of EN treatment through nasal catheters.
- EN treatment time ≥ 7 days.
- Age>18 years old.
- Acute Physiological and Chronic Health Score II (APACHE II) at admission.

### Exclusion criteria:

- Inflammatory bowel disease, irritable bowel syndrome and other diseases that can lead to diarrhea.
- Onset of diarrhea before the initiation of EN.
- Complications such as intestinal ischemia, perforation, gastrointestinal bleeding, nausea and vomiting occur during EN.
- Malignant tumors.
- Concomitant mental illness.

### Data collection:

- Age, gender, Body mass index (BMI), education level.
- APACHE II score, disease type, hypertension, diabetes, Hypoproteinemia, fasting time at admission.
- Abdominal surgery in the past five days, mechanical ventilation, ICU stay time, antibiotic use>2 weeks, dilution of EN preparations, EN infusion method, daily dosage of EN preparations, EN infusion rate, gradual increase of EN preparations, oral potassium preparations, and albumin levels.

Diarrhea was defined as the occurrence of three or more loose or watery stools within a 24-hour period, accompanied by an obvious change from the patient’s usual bowel habit. Only diarrhea episodes that occurred after the initiation of enteral nutrition in the ICU were considered EN-related diarrhea and included as outcome events in this study. All cases were identified based on nursing documentation and physician confirmation using a standardized diagnostic protocol applied uniformly across the ICU.

### Statistical analysis:

The statistical software used is SPSS 22.0 and R software version 4.0.0. Measurement data that conformed to the normal distribution were expressed as (*χ̅*±*S*). The inter group comparison used independent sample *t* test. Data that did not meet the normal distribution were expressed by interquartile range (IQR). The inter group comparison used Mann-Whitney *U* test. Counting data were expressed as n (%), and Chi-squared test was used for comparison between groups. A binary logistic regression model was used to analyze the risk factors for EN-related diarrhea. First, univariate logistic regression was performed for all candidate variables, and those with p < 0.05 were entered into a multivariate logistic regression model using a forward stepwise approach. To avoid multicollinearity, the variance inflation factor (VIF) was calculated for each predictor; all VIF values were <2, indicating no significant collinearity. Missing data were handled using a complete case analysis strategy. Based on the final multivariate model, a nomogram was constructed using the “rms” package in R software (version 4.0.0), with predictor weights directly derived from the β coefficients (log odds ratios) of the logistic regression. Model discrimination was assessed by calculating the area under the receiver operating characteristic curve (AUC), and calibration was evaluated by plotting a calibration curve using the Bootstrap method with 1,000 resamples. Internal validation of the nomogram was also performed with Bootstrap resampling. A two-sided p value < 0.05 was considered statistically significant.

## RESULTS

A total of 402 patients were included in this study, of which only 120 patients experienced postoperative EN diarrhea (diarrhea group), while 282 patients did not experience EN diarrhea (non-diarrhea group).

There were statistically significant differences in the hypoproteinemia, fasting time, abdominal surgery in the past five days, antibiotic use>2 weeks, infusion rate of EN solution, gradual increase of EN preparation, oral potassium preparation, and albumin levels between the two groups (*p*<0.05) Table-I.

**Table-I:** Univariate analysis.

Influencing factor	Non diarrhea group (n=282)	Diarrhea group (n=120)	χ^2^/t/Z	p-Value
Age (Years)	74.78±5.81	74.20±5.49	0.926	0.355
Gender, n (%)			0.138	0.711
Male	140 (49.65)	62 (51.67)		
Female	142 (50.35)	58 (48.33)		
BMI (kg/m^2^)	26.25±2.31	26.49±2.11	-0.972	0.332
Degree of education, n (%)			0.006	0.997
Junior high school and below	76 (26.95)	32 (26.67)		
Technical secondary school/high school	127 (45.04)	54 (45.00)		
College degree or above	79 (28.01)	34 (28.33)		
APACHEⅡ score on admission (score), M(IQR)	20 (17-22)	19 (17-22)	-	0.294
Disease type, n (%)			0.483	0.923
Respiratory system	123 (43.62)	52 (43.33)		
Nervous system	76 (26.95)	32 (26.67)		
Digestive system	75 (26.60)	31 (25.83)		
Other	8 (2.84)	5 (4.17)		
Hypertension (yes), n (%)	56 (19.86)	24 (20.00)	0.001	0.974
Diabetes (yes), n (%)	70 (24.82)	31 (25.83)	0.046	0.831
Hypoproteinemia (yes), n (%)	55 (19.50)	58 (48.33)	36.623	<0.001
Fasting time, n (%)			22.618	<0.001
<5d	247 (87.59)	81 (67.50)		
≥5d	35 (12.41)	39 (32.50)		
Mechanical ventilation (yes), n (%)	130 (46.10)	57 (47.50)	0.066	0.797
ICU stay time(d)	7 (6,8)	7 (6,8)	-0.333	0.739
Antibiotic use > 2 weeks (yes), n (%)	41 (14.54)	41 (34.17)	19.972	<0.001
Enteral nutrition preparation diluted (yes), n (%)	98 (34.75)	36 (30.00)	0.855	0.355
Enteral nutrient solution infusion mode, n (%)			0.689	0.407
Continuous infusion	142 (50.35)	55 (45.83)		
Intermittent infusion	140 (49.65)	65 (54.17)		
Daily dosage of enteral nutrition preparation, n (%)			6.175	0.013
<1200 ml/day	215 (76.24)	77(64.17)		
≥1200 ml/day	67 (23.76)	43 (35.83)		
Enteral nutrient infusion rate, n (%)			25.002	<0.001
<100ml/h	224(79.43)	66(55.00)		
≥100ml/h	58(20.57)	54(45.00)		
Enteral nutrition preparation increments gradually (yes), n (%)	165 (58.51)	34 (28.33)	30.667	<0.001
Oral potassium preparation (yes), n (%)	76 (26.95)	74 (61.67)	43.374	<0.001
Albumin level, n (%)			30.871	<0.001
<35g/L	232 (82.27)	67 (55.83)		
≥35g/L	50 (17.73)	53 (44.17)		
Abdominal surgery in the last 5 days (yes), n (%)	19 (6.74)	21 (17.50)	10.882	0.001

After assigning values to the factors with *p*<0.05 in the above univariate analysis as independent variables (Table-II), multifactor logistic regression analysis was carried out. Hypoproteinemia, abdominal surgery in the past five days, fasting time>5 days, antibiotic use>2 weeks, no gradual increase in EN preparations, oral potassium preparations, EN fluid infusion rate ≥ 100ml/h, and albumin level ≥ 35g/L were identified as risk factors for EN diarrhea in ICU patients (Table-III).

**Table-II:** Assignment of each factor.

Influencing factor	Assignment
Hypoproteinemia	No=0；Yes=1；
Fasting time	<5d=1；≥5d=2；
Antibiotic use > 2 weeks	No=0；Yes=1；
Daily dosage of enteral nutrition preparation	<1200 ml/day=1；≥1200 ml/day=2；
Enteral nutrient infusion rate	<100ml/h=1；≥100ml/h=2；
Enteral nutrition preparation increments gradually	No=0；Yes=1；
Oral potassium preparation	No=0；Yes=1；
Albumin level	<35g/L=1；≥35g/L=2；
Abdominal surgery in the last 5 days	No=0；Yes=1；

**Table-III:** Results of multivariate Logistic regression analysis.

	B	S.E.	Wald χ^2^	p-Value	OR	95%CI	
						Lower limit	Upper limit
Hypoproteinemia	1.281	0.299	18.373	<0.001	3.599	2.004	6.464
Fasting time	1.260	0.330	14.583	<0.001	3.527	1.847	6.735
Antibiotic use > 2 weeks	0.815	0.330	6.088	0.014	2.260	1.183	4.319
Daily dosage of enteral nutrition preparation	0.477	0.305	2.450	0.118	1.611	0.887	2.928
Enteral nutrient infusion rate	1.110	0.296	14.099	<0.001	3.036	1.700	5.419
Enteral nutrition preparation increments gradually	-1.266	0.292	18.773	<0.001	0.282	0.159	0.500
Oral potassium preparation	1.504	0.288	27.268	<0.001	4.502	2.559	7.918
Albumin level	1.097	0.300	13.378	<0.001	2.996	1.664	5.395
Abdominal surgery in the last 5 days	1.260	0.423	8.871	0.003	3.527	1.539	8.083

**Fig.1:**
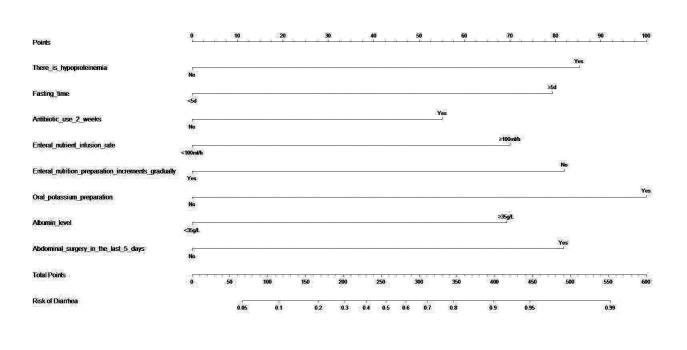
Nomogram Model.

**Fig.2:**
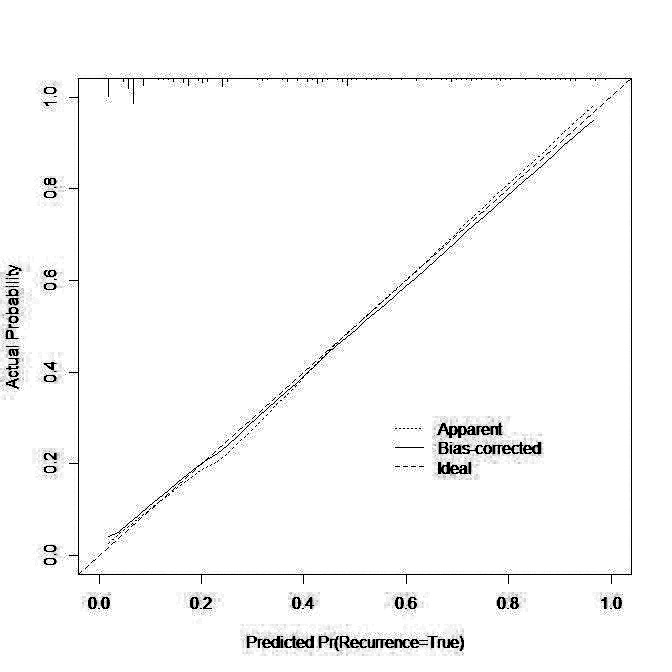
Correction Curve.

**Fig.3:**
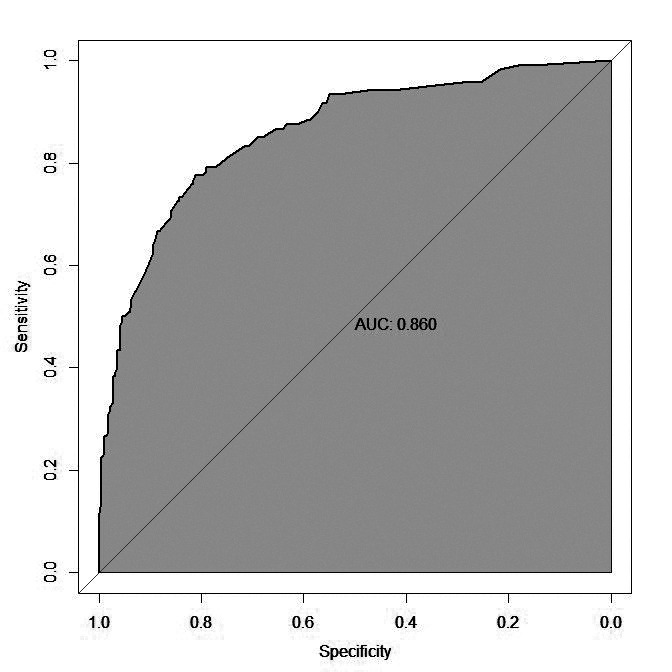
Receiver operating characteristic Analysis.

The nomogram was then drawn using significant influencing factors that were identified in the multivariate logistic regression model including hypoproteinemia, fasting time, antibiotic use>2 weeks, EN liquid infusion rate, gradual increase of EN preparations, oral potassium preparations, albumin level, and abdominal surgery in the last five days (Fig.1). Internal validation of the prediction nomogram model was conducted using the Bootstrap method, and basically fitted the ideal model. The calibration curve showed good consistency between actual observations and nomogram predictions (Fig.2). ROC curve analysis of the value of the model in predicting diarrhea in ICU patients during EN treatment showed that the AUC of the model was 0.860 (95% CI: 0.820-0.901), indicating a certain predictive value. When the optimal cut off value was selected, sensitivity and specificity of the model were 77.5% and 81.2%, respectively. Indicating that the Nomogram model has good predictive performance (Fig.3).

## DISCUSSION

The results of our study showed that diarrhea during the ICU EN treatment occurs with the incidence of 29.85% (120/402). Hypoproteinemia, abdominal surgery in recent five days, fasting time > 5 days, antibiotic use > 2 weeks, no gradual increase of EN preparations, oral potassium preparations, EN liquid infusion rate ≥ 100ml/h, albumin level ≥ 35g/L were all identified as risk factors for EN diarrhea in the ICU setting.

The biological plausibility of these findings can be supported by known pathophysiological mechanisms. Hypoproteinemia can reduce plasma oncotic pressure, leading to intestinal mucosal edema and increased permeability. This may impair villus function and hinder fluid reabsorption, ultimately contributing to diarrhea.^8^ Recent abdominal surgery may impair gastrointestinal motility due to surgical trauma, neural inhibition, or peritoneal inflammation, leading to transient ileus and nutrient malabsorption.^9^ Long-term antibiotic use disrupts gut microbial diversity, reduces protective commensal flora, and promotes overgrowth of pathogenic bacteria (e.g., Clostridium difficile), which increases the risk of antibiotic-associated diarrhea.^10^ These mechanisms provide physiological justification for the model’s identified predictors and reinforce their clinical relevance.

EN support is the preferred feeding method for ICU patients with normal gastrointestinal function. It can provide sufficient nutritional support for patients, and is associated with relatively ideal tolerance.^11^ However, complications, such as EN diarrhea may exacerbate malnutrition, interrupt nutritional support, and increase mortality rates.^12^,^13^ Our study identified hypoproteinemia as one of the risk factors of this complication in ICU patients who receive enteral support. Our results are similar to the study of Zeng et al.^14^ that showed that patients with hypoproteinemia have a higher risk of enteric nutrition-related diarrhea. Hypoproteinemia can reduce the permeability of plasma colloid, cause intestinal mucosal edema, reduce the absorption function of intestinal villi, increase the osmotic pressure inside and outside of blood vessels, lead to a large amount of liquid entering the intestinal cavity, imbalance of intestinal flora, and ultimately, cause diarrhea.^15^

The history of abdominal surgery in the past five days was also identified as a risk factor in our study. Studies show that as abdominal surgery can cause nerve damage of the gastrointestinal tract, gastrointestinal peristalsis of patients returns to normal functioning only 3-4 days after the surgery. Therefore, this type of surgical patients has lower gastrointestinal function and lower tolerance of EN support, making thyme highly susceptible to EN diarrhea.^16^,^17^

This study showed that fasting time > 5 days was associated with higher risk of diarrhea in ICU patients who receive enteral support. Our results are consistent with the research results of Darwin A et al.^18^ that showed that longer the fasting time before EN support increases the risk of diarrhea during the EN therapy. Long-term fasting negatively affects physical and chemical balance of patient’s intestinal mucosa, causing “disuse dysfunction”, absorption disorders and diarrhea.^19^

Antibiotic use > 2 weeks in our study was associated with higher risk of EN diarrhea. Previous research demonstrated the adverse impact of long-term and massive use of antibiotics on gut microbiota, leading to diarrhea, which is in agreement with our results.^20^ Improper administration of large amounts of EN preparations instead of gradually increased dosages was another risk factor, identified in our study. Our results are similar to the research results of Kim et al.^21^ that demonstrated that when the dose of EN is not increased step by step, a large amount of extracellular fluid is absorbed, stimulating intestinal peristalsis, and causing diarrhea.

Previous studies showed that long-term oral administration of potassium preparations cannot be fully absorbed by the small intestine, leading to intestinal liquid retention and diarrhea.^22^ Consistently with this observation, our study identified oral potassium preparation as a risk factor of EN diarrhea.

Infusion rate of enteral nutrient solution ≥ 100ml/h led to higher risk of diarrhea in ICU patients in our study. This result is similar to the study by Qu et al.^23^ When the infusion rate of nutrient solution is too fast, a large amount of nutrient solution enters the intestines in a short period of time, reducing intestinal digestion and absorption functions, and leading to diarrhea.

Albumin level ≥ 35g/L was identified as a risk factor of diarrhea, which is similar to the results of Kanner et al.^24^ that showed that low albumin level leads to lower plasma oncotic pressure. As a result, water is transferred to the peripheral tissues, leading to the edema of intestinal mucosa, nutrition absorption dysfunction, and diarrhea.

While, studies of Shi et al.^25^ and Batassini et al.^26^ showed that age and APACHE II score at admission were also risk factors for EN diarrhea in ICU, these factors were not included in the regression equation of this article. This discrepancy may be related to sample size selection bias, and calls for further research.

The results of our study suggest that implementing EN support for patients in ICU clinical practice requires careful control of dosage and rate of nutrition. For patients who require antibiotics, pathogen detection and drug sensitivity tests should be carried out to select the appropriate antibiotic treatment in combination with measures to preserve gut microbiota. Patients with long-term fasting and hypoproteinemia may benefit from parenteral nutrition or albumin infusion. During the treatment, care should be taken to ensure aseptic administration of EN to avoid contamination of nutrient solution and reduce the risk of diarrhea.

Beyond statistical accuracy, the nomogram model developed in this study aims to serve as a practical tool for individualized clinical decision-making in ICU enteral nutrition management. It can be implemented during ICU admission or early nutritional assessment to stratify patients by diarrhea risk. For patients with high nomogram scores, clinicians may consider initiating EN at a lower infusion rate (e.g., < 60 ml/h), adopting a stepwise escalation protocol, avoiding hyperosmolar or potassium-rich formulations, and monitoring serum albumin levels closely. In addition, strategies such as timely albumin supplementation and careful control of antibiotic duration (e.g., avoiding unnecessary prolonged use) may help mitigate risk. Integrating the model into ICU electronic health records or bedside tools could facilitate early screening and intervention. While internal validation demonstrated strong predictive performance (AUC = 0.860), further prospective interventional studies are warranted to evaluate whether nomogram-guided management reduces diarrhea incidence and improves clinical outcomes in critically ill patients.

### Strength of this study:

This study incorporates a comprehensive set of clinically relevant predictors and presents statistically stable results, ensuring a degree of representativeness and reliability. The findings provide meaningful reference value for the prevention and management of enteral nutrition (EN)–related diarrhea in ICU patients. Our nomogram model enables individualized risk estimation for EN-related diarrhea during ICU hospitalization, allowing for early identification of high-risk patients and timely implementation of targeted preventive strategies. Compared with existing models or scoring tools for gastrointestinal complications in ICU populations, our model offers several novel and practical advantages. First, it is specifically designed to predict EN-related diarrhea, rather than generalized gastrointestinal intolerance or diarrhea in broader cohorts. Second, it integrates both baseline risk factors (e.g., hypoproteinemia, recent abdominal surgery, prolonged fasting, extended antibiotic use) and modifiable nutrition management parameters (e.g., EN infusion rate, stepwise titration, oral potassium use), which are readily accessible and actionable in clinical practice. Third, the visualized nomogram format facilitates intuitive, bedside application, supporting individualized intervention planning. Finally, the model demonstrated good internal performance, with high discrimination (AUC = 0.860, 95% CI: 0.820–0.901) and calibration.

### Limitations:

First, it was a single-center, retrospective analysis with a relatively small sample size, which may introduce selection and information bias and limit the generalizability of the findings. Although we applied strict inclusion and exclusion criteria and extracted data from standardized electronic medical and nursing records—cross-validated by two independent researchers and a third senior reviewer—retrospective designs inherently carry risks of bias. Missing data were handled through complete case analysis to ensure analytic integrity. Second, although the model demonstrated good internal validity through Bootstrap calibration and excellent discrimination (AUC = 0.860), neither cross-validation nor external validation was performed. This limits our ability to assess model robustness and applicability across diverse populations. Future studies should incorporate k-fold cross-validation and external validation using large, prospective, multicenter datasets across various ICU types (e.g., surgical, medical, neurological) to enhance generalizability. Third, some clinically important variables were not included due to either lack of significance or unavailability in the dataset. Variables such as age, comorbidities (e.g., diabetes, hypertension), ICU length of stay, and APACHE II score were included in the univariate analysis but excluded from the final multivariate model due to non-significance (p > 0.05). Nonetheless, these factors may hold clinical relevance. Age-related gastrointestinal dysfunction, polypharmacy in patients with chronic diseases, and disease severity may contribute to diarrhea risk and warrant further investigation in future studies with larger and more heterogeneous cohorts. Furthermore, this study did not incorporate subgroup analyses across ICU disease types, which limits the granularity of the findings.

## CONCLUSION

The nomogram model constructed based on the above factors demonstrated good predictive performance (AUC = 0.860). It can be applied at ICU admission or during early nutrition planning to screen for patients at high risk of EN-related diarrhea. For those with high-risk scores, clinicians may consider initiating enteral nutrition at a slower infusion rate, avoiding high-osmolality or potassium-rich formulas, using a stepwise dose-escalation strategy, and closely monitoring patients with hypoalbuminemia or recent abdominal surgery. These targeted strategies may help reduce the incidence and severity of diarrhea in ICU settings. While promising, the nomogram should be used as a decision-support tool rather than a replacement for clinical judgment. Future multicenter prospective studies are needed to externally validate this model and to further evaluate the effectiveness and clinical impact of nomogram-guided interventions in diverse ICU populations.

### Recommendations:

Future research should stratify patients by diagnosis to explore condition-specific risk profiles. Lastly, the dataset lacked important predictors such as immune status (e.g., lymphocyte subsets, immunosuppressive therapy), gut microbiota characteristics, and certain biochemical markers, which may affect diarrhea development. The integration of these variables, along with the application of advanced modeling techniques such as machine learning or dynamic time-dependent models, may improve the nomogram’s performance and clinical utility.

### Authors’ contributions:

**DZ and TC:** Literature search, study design and manuscript writing.

**YH, QH and SJ:** Data collection, data analysis and interpretation, Critical review.

**DZ and TC:** Manuscript revision and validation and is responsible for the integrity of the study.

All authors have read and approved the final manuscript.
